# Challenges and applications in generative AI for clinical tabular data in physiology

**DOI:** 10.1007/s00424-024-03024-w

**Published:** 2024-10-17

**Authors:** Chaithra Umesh, Manjunath Mahendra, Saptarshi Bej, Olaf Wolkenhauer, Markus Wolfien

**Affiliations:** 1 Institute of Computer Science, Department of Systems Biology and Bioinformatics, University of Rostock, Rostock, Germany; 2 School of Data Science, Indian Institute of Science Education and Research (IISER), Thiruvananthapuram, India; 3 Leibniz-Institute for Food Systems Biology, Technical University of Munich, Freising, Germany; 4 Faculty of Medicine Carl Gustav Carus, Institute for Medical Informatics and Biometry, TUD Dresden University of Technology, Dresden, Germany; 5 Center for Scalable Data Analytics and Artificial Intelligence (ScaDS.AI), Dresden, Germany

**Keywords:** Generative AI, Tabular data, Physiological applications, Data privacy

## Abstract

Recent advancements in generative approaches in AI have opened up the prospect of synthetic tabular clinical data generation. From filling in missing values in real-world data, these approaches have now advanced to creating complex multi-tables. This review explores the development of techniques capable of synthesizing patient data and modeling multiple tables. We highlight the challenges and opportunities of these methods for analyzing patient data in physiology. Additionally, it discusses the challenges and potential of these approaches in improving clinical research, personalized medicine, and healthcare policy. The integration of these generative models into physiological settings may represent both a theoretical advancement and a practical tool that has the potential to improve mechanistic understanding and patient care. By providing a reliable source of synthetic data, these models can also help mitigate privacy concerns and facilitate large-scale data sharing.

## Introduction

In data analysis, real-world data often has missing values, which limits the ability to draw conclusions and insights from such data. Traditionally, these missing values are filled out using data imputation methods, which fill in synthetic values. Over time, these methods have evolved from imputing the part of the data to creating linked tables. This review summarizes the challenges and advancements of generative techniques capable of synthesizing entire tabular data.

### The importance of tabular data generation

Commonly, clinical data is stored using tabular database frameworks [10, 26]. They are simple to maintain, analyze, and interpret [10]. Columns indicate the features or attributes related to each row’s records or observations in a tabular data structure. The data for each record and attribute are represented as a value in each table cell. This structure makes it possible to store, retrieve, and analyze data in an organized and systematic way [10]. There are several methods for gathering tabular data. A questionnaire survey, patient-reported data, genetic information, proxy or informant data, a review of ambulatory or hospital medical records, and a collection of biological samples are the most frequently used data-gathering methods among many others in clinical research [47, 64].

Mostly, tabular data is not stored as single tables; instead, large databases (e.g., in a data warehouse or data lake) are built to store data [42]. For efficient storage of data, databases use multiple linked tables. This helps to avoid redundancy in data storage and contributes to the robustness of the database. “Normalization” is used in Database Management Systems to break down a large volume of information into smaller bits, where each bit of information contributes to a single table in a database [55]. Some examples of clinical databases include MIMIC-III, MIMIC-IV, and the National Inpatient Sample (NIS) database [17, 29].

**Fig. 1:**
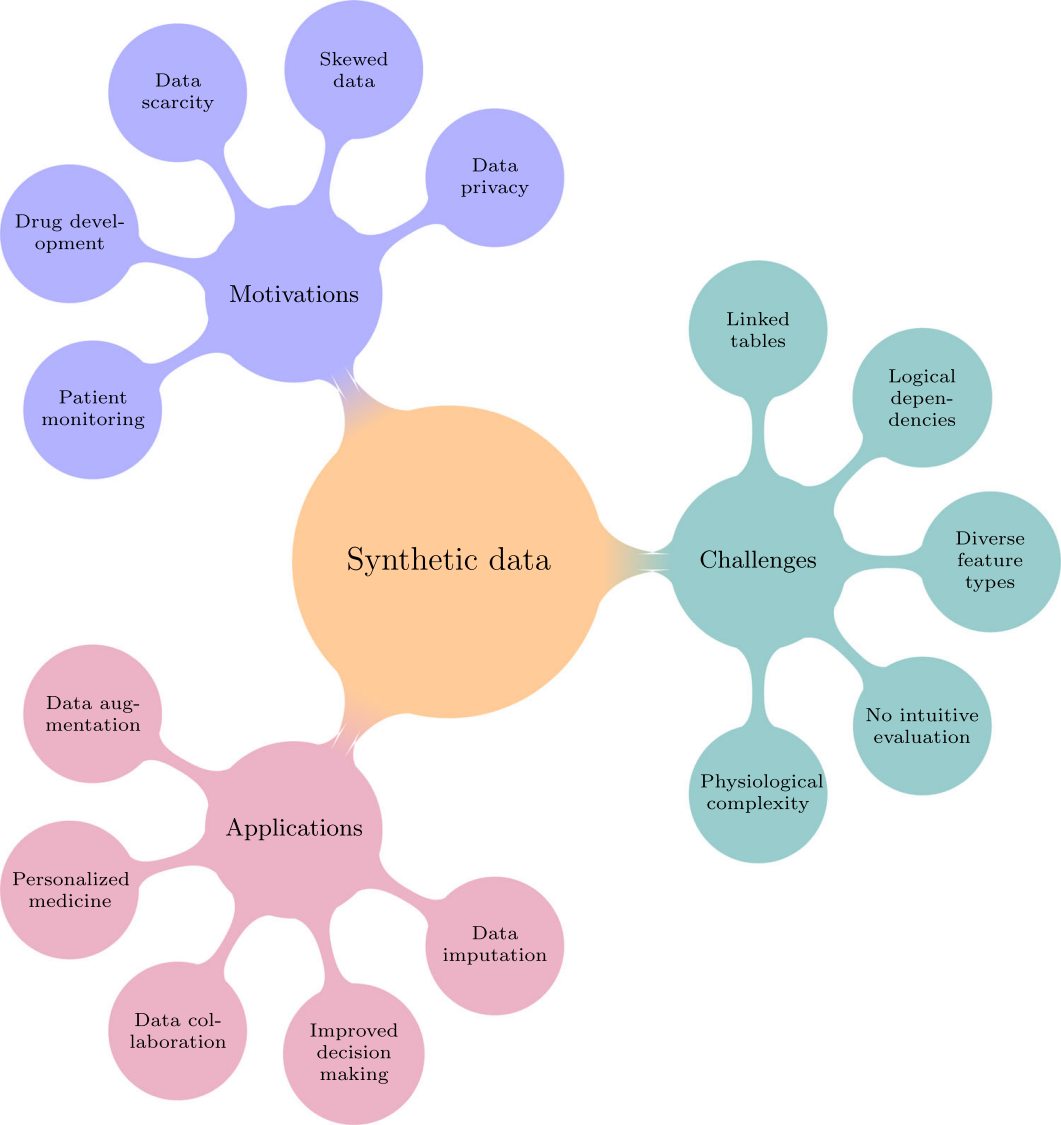
Overview of motivations, challenges, and applications of synthetic data

From a practical perspective, query languages like MySQL are often used to extract information from multiple tables of such databases. For example, an SQL query may retrieve the names and sexes of patients by joining the patients and medical records tables on their patient IDs and filtering for females with a “pregnant” status based on their medical records. The combined information across several tables in a database can subsequently form a single dataset that might be used for further analysis. Tables storing such combined information can contain a lot of dependencies among the attributes. For example, suppose there is a column in the table containing an attribute for the sex of the patient and another attribute for the patient’s pregnancy status. It is logically obvious that a patient whose sex is male cannot have a positive pregnancy status.

Inspired by the human brain’s structure, artificial neural networks are interconnected layers of nodes that process information like neurons. This technology has evolved from basic networks to deep learning and generative models. Generative models mimic how our brains learn by analyzing existing data to create new data, similar to how we use past experiences to imagine new situations. These techniques allow us to generate more realistic data, leading to better insights and solutions in science and medicine (Fig. 1).

### Why is synthetic data sharing in medicine useful?

Concerning data privacy, a review on synthetic data in healthcare by Gonzales et al. [22] points out that most health data are not readily available because they contain confidential information about individuals. Identifiable records can also not be easily shared as organizations need to comply with certain regulations, such as the Health Insurance Portability and Accountability Act of 1996 (HIPAA) in the USA [60]. When real data is unavailable, especially in the clinical domain, the idea of synthetic data as a “proxy” of real data has taken shape over the past few years [3].

Even though synthetic data generation started with image-based datasets, recently, several models have been developed for tabular synthetic data generation [1, 35, 51, 65, 70]. An interesting dilemma pointed out in Kaabachi et al. is that a synthetic dataset that most closely mimics the original dataset is likely also to be most useful, but at the same time, provide less privacy protection. If the synthetic data is very close to real data, one can easily trace it back to real data. On the other hand, a synthetic dataset that is very different from the original data will provide strong protection but likely less utility [31]. This also points out that for different practical tasks, the strategy to be adapted for synthetic data generation could be different, generating a lot of room for research. Researchers are also vigilant in analyzing the reliability of synthetic data in several scenarios, such as a controlled clinical study [18]. Several studies in the healthcare domain exist that replicate case studies originally performed on health-related data using alternative synthetically generated data [2, 15].

Shi et al. [53] adapted ADS-GAN for generating fictitious patient data and employed a neural network to predict treatment outcomes. They created a realistic synthetic dataset comprising over 580, 000 hypertension patients’ data, including multi-year medical histories, to evaluate over ten treatments’ effects on blood pressure outcomes. They further used the privacy metric, which estimates the probability of actual patients being identified is 0.008%, ensuring that the synthetic data maintains patient anonymity [53]. In addition to privacy, they also show that the distribution of synthetic data is similar to that of real data based on distance metrics. Yale et al. [66] propose another end-to-end generative architecture, Health-GAN, to relate privacy-preserving synthetic data.

Gonzales et al. [22] identify several other use cases of synthetic data generation in healthcare, such as simulation and prediction research; hypothesis, methods, and algorithm testing; epidemiology/public health research; health IT development; education and training; public release of datasets; and linking data.

One can, therefore, conclude that current generative models can create high-quality synthetic data that mirrors real patient data, enabling researchers to access and share data that drives decision-making while preserving patient privacy. This synthetic data can fill gaps in existing datasets, ensuring more comprehensive and diverse data for analysis. In the following section, we will explore in more detail how this can impact the field of physiology research.

## Challenges of tabular data generative approaches

Clinical and bio-medical data are essential components of healthcare systems, and they typically comprise information about patients’ demographics, socioeconomic conditions, medical history, etc. [47]. Machine learning (ML) algorithms are often employed to make clinical decisions from such data, and several State-Of-The-Art (SOTA) ML algorithms are available for this purpose. However, in some cases, data is unavailable for clinical decision-making due to privacy protocols or data scarcity. One possible solution is generating synthetic samples that follow the same statistical properties as real data. Generating tabular data poses several challenges, which are also relevant to physiology. In the following, there is an explanation of the difficulties in modeling synthetic tabular data and their connection to physiology.

### Diverse feature types and multi-modality

Clinical tabular data has different types of features, namely categorical and continuous [7]. Continuous features can take any value within a specified range, like the height of a patient. Imagine the frequency distribution of a feature or attribute with more than one peak. The peaks of a frequency distribution represent the values of an attribute that a data point is most likely to adapt. Each peak is called a mode, and an attribute with a frequency distribution with multiple peaks is said to follow a multi-modal distribution. If we think in a multivariate or multi-attribute scenario, a multi-modality of attributes implies that there might be different groups within the same dataset. Multi-modality in features can make generating synthetic data that matches the original distribution is hard.

In addition, categorical features increase the complexity of generative models because they represent discrete and non-numeric values. Categorical features are usually divided into nominal and ordinal data. Nominal data refers to features like the sex of a patient, to which no sense of order is attached. In contrast, ordinal data refers to features like the level of alcohol consumption, to which a sense of order is associated [6]. Usually, continuous probability distributions are used to model synthetic data space. For example, a variational autoencoder learns a parameterized latent distribution, usually Gaussian [1]. Categorical variables with discrete distributions might be difficult to integrate with such modeling paradigms.

Derived physiology challenges can include, for example,

- **Modeling complex biological systems:** Physiological systems are complex and often difficult to model accurately. Generative AI can help create more nuanced models of these systems, leading to a better understanding of physiological processes.
- **Personalized medicine:** There is a growing need for personalized approaches in medicine. Generative AI can aid in developing personalized models of physiological responses, predicting how individuals might react to medications or treatments based on their unique physiological makeup.

### Class imbalance

Dealing with data imbalance or skewness is a common problem when working with clinical datasets, as specific classes are frequently underrepresented [19, 51]. For example, in the case of rare diseases like cystic fibrosis, information about the healthy group is more than that of the diseased group. This imbalance can cause problems when training generative models. Such models tend to generate data from the more abundant or frequent values of attributes while ignoring less abundant but contextually important ones. This is known as the problem of mode collapse [4], meaning that the synthetic data is generated only from certain modes of higher frequency. Consequently, developing reliable predictive models that can learn from skewed synthetic data becomes difficult.

Derived physiology challenges include, for example,

- Filling data gaps in rare diseases: Obtaining sufficient data for research on rare diseases can be challenging. Generative AI can create synthetic data sets that mimic the characteristics of rare diseases, facilitating research without the need for large, real-world datasets.
- Improving medical imaging and diagnostics: AI can generate realistic medical images for training purposes or augment existing datasets, improving the ability of diagnostic algorithms to detect and interpret medical conditions.
- Understanding disease progression: AI models can help understand and predict disease progression, particularly those with complex physiological impacts or time-series information, like diabetes or heart disease. This can lead to earlier interventions and more effective treatment plans.

### Dependencies between the attributes

Tabular data exhibits relationships among its features, unlike image and text data. Preserving relationships within the synthetic samples is essential, like ensuring consistency between features such as “gender” and “pregnancy status.” This is crucial in healthcare or demographics, where data integrity matters the most. Establishing metrics to assess dependency preservation and addressing challenges like capturing implicit dependencies are essential for improving the quality of synthetic tabular data. There are various synthetic data generation models for tabular data, but they do not focus on dependency preservation.

Derived physiology challenges can include, for example,

- **Drug development and discovery:** AI can generate novel molecular structures for potential drugs, simulate their effects on the human body, and predict side effects, significantly speeding up drug discovery.
- **Bridging in vitro and in vivo studies:** There is often a disconnect between laboratory (in vitro) studies and real-world (in vivo) observations. Generative AI can help bridge this gap by simulating how cellular or molecular processes observed in the lab might play out in a living organism.
- **Simulation of environmental and lifestyle impact:** Generative AI can simulate the long-term effects of environmental factors or lifestyle choices on human physiology, aiding in public health planning and preventive healthcare strategies.

## Technical description of state-of-the-art generative models

Understanding the technical description of state-of-the-art generative models is also essential for non-computational experts. These models, which include advanced techniques like neural networks and deep learning algorithms, can simulate biological processes and generate realistic synthetic data. For physiologists, this knowledge enables them to leverage cutting-edge AI tools to enhance their research, improve the accuracy of their studies, and develop innovative solutions to the aforementioned physiological challenges. By staying informed about these technological advancements, physiologists can better interpret and apply AI-generated data, leading to more precise and impactful scientific discoveries [46].

In the realm of generating synthetic tabular data, researchers have explored various approaches to replicate the underlying distribution of real-world datasets. Notable among these are generative adversarial networks (GANs), variational autoencoders (VAEs), diffusion models, convex space generators, and large language models (LLMs). Each of these methods offers a unique perspective on the generation process based on different data characteristics and modeling objectives [20, 69], which are briefly discussed in the following:

*GANs* are a type of deep learning model that has gained much attention recently. GANs train two models, a generator and a discriminator, in a zero-sum game setting. The generator learns to create new data samples similar to the real ones, while the discriminator learns to distinguish between real and fake samples. The idea behind GANs is to estimate the probability distribution of real data samples and to generate new samples from that distribution. GANs have shown great potential for a wide range of applications, including image and vision computing, speech and language processing, and many others [20, 23, 65, 70, 71].

**Fig. 2:**
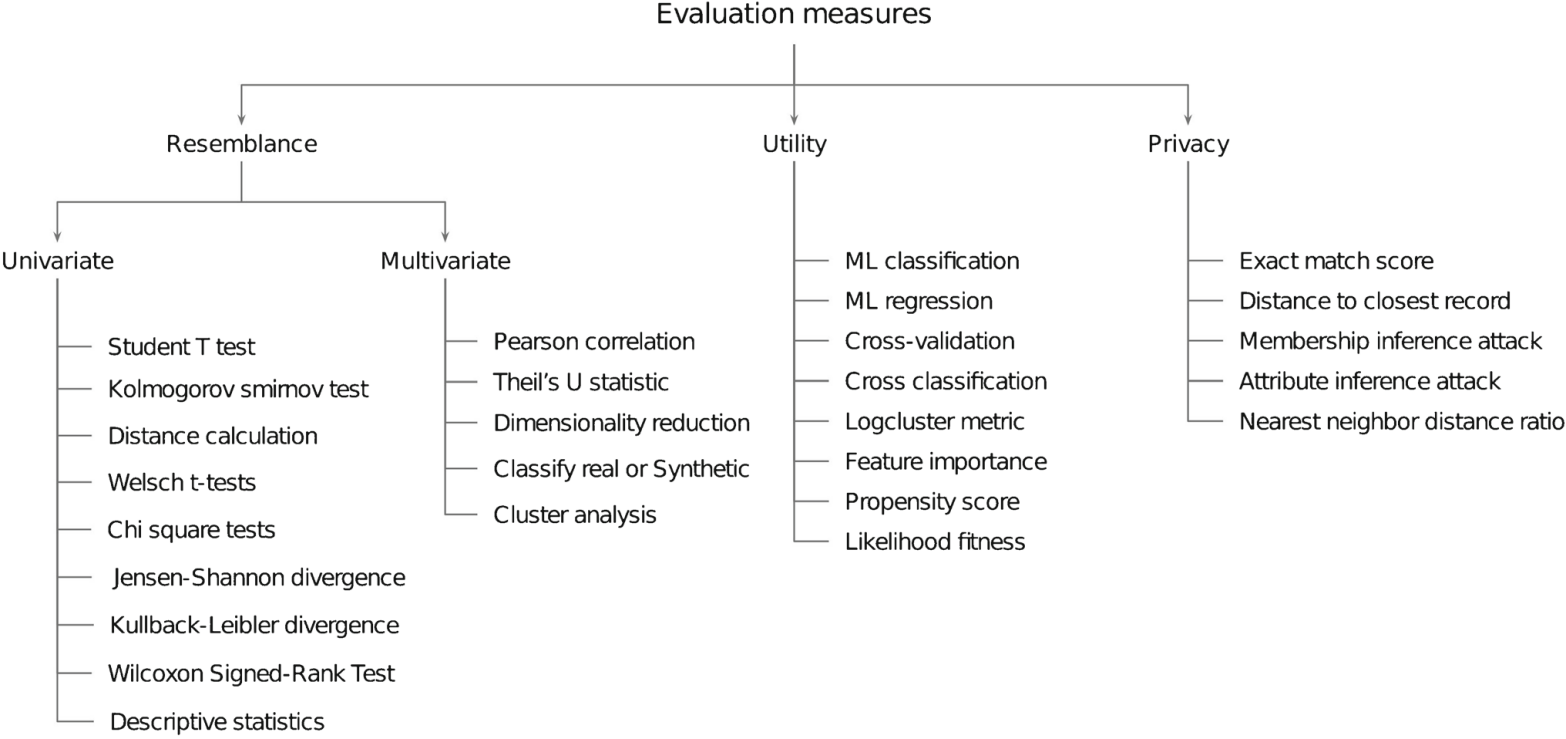
Organizational chart depicting commonly used evaluation measures for synthetic tabular data

*VAEs* are a type of generative models that employ variational Bayesian inference to approximate the probability density of a dataset. VAEs consist of two key components: an encoder and a decoder. The encoder takes in the input data and passes it through a series of layers that gradually reduce its dimensionality. The encoder output is a compressed representation of the input data mapped to a latent space. The decoder then uses variational inference to sample from the latent space and generate a reconstruction of the input data. By training the encoder and decoder networks together, VAEs can learn to generate new data points that are similar to the ones in the original dataset [34, 48, 61]. With advancements in representation learning, these models can efficiently handle mixed-type longitudinal data, as demonstrated by innovations like EHR-M-GAN, which generates synthetic electronic health records (EHR) [36]. Such advancements enable more comprehensive and precise modeling of patient data over time, fostering improved predictive analytics.

*Diffusion models* are likelihood-based generative models that handle the data through forward and reverse Markov processes. In the forward process, noise is gradually added to the data distribution by sampling noise from predefined distributions with varying variances. Conversely, the reverse process denoises a latent variable and allows for the generation of new data samples. Since the distributions are often unknown, they are approximated by a neural network with learnable parameters [49, 72].

*Convex space learning* has been developed to generate synthetic data within the data space where they share similar characteristics and are closely related. This is achieved through a deep cooperative learning approach involving two neural networks: the generator and discriminator networks. During training, the generator network produces synthetic data within the convex space, while the discriminator network evaluates the quality of the generated samples. This technique utilizes convex coefficients that are learned during the training process to generate synthetic data that is similar to the real-world data [52].

*LLMs* are a type of deep learning architecture, typically employing an encoder-decoder structure, that was initially developed to solve general-purpose natural language processing (NLP) tasks [59]. The encoder acts as the initial processing unit and analyzes the input text. This analysis focuses on capturing the essential meaning and context within the input. The processed information is then passed on to the decoder. The decoder utilizes this encoded representation to generate the model’s response. This response can take various forms, such as a translation of the original text, a narrative continuation, or the creation of entirely new yet coherent, synthetic data with high levels of realism. Due to its adaptability, it is been widely used across various fields, including healthcare. It has proven effective in analyzing medical imaging, structured and unstructured EHR, social media, physiological signals, and biomolecular sequences [41].

## Evaluation measures

To ensure the continued success of AI models, it is essential to constantly enhance evaluation methods to determine the effectiveness and reliability of synthetic data used to represent real-world data (Fig. 2). Unfortunately, there are currently no standard measures for evaluating tabular synthetic data. Each author has used a different set of measures to evaluate their models. We have attempted to compile commonly used evaluation metrics and categorize them into three main dimensions: resemblance, utility, and privacy.

- *Resemblance/fidelity* refers to how similar the synthetic data is to the real data regarding statistical properties and relationships between features. This similarity is crucial for the synthetic data to be a valid replacement for the real data in various applications. Statistical tests like the Student’s *t*-test, Kolmogorov-Smirnov test, Mann Whitney U-test for continuous features [27] and KL divergence, Chi2 test [16], Hellinger distance, and Jensen-Shannon divergence for categorical features are used to compare probability distributions of features in real and synthetic data [19]. Correlation metrics like Pearson correlation for continuous features, Spearman rank correlation for ordinal features, and Chi-squared test for feature independence are used to assess the relationship between features. Similar correlation coefficients between real and synthetic data suggest a reasonable resemblance.
- *Utility* is how well the synthetic data can perform on various machine learning tasks and for subsequent downstream data analyses, e.g., survival curves. Utility measures for synthetic data are determined by the performance of synthetic data for any given task, such as classification or regression. Jordon et al. state that fidelity is often considered alongside utility [30]. Metrics like cross classification, log cluster metric, propensity score, data correlation [19, 21, 43], and classification performance are some of the utility measures. If scores obtained by the models trained on real and its corresponding synthetic data independently produce similar scores for the above-mentioned measures, it indicates an actionable utility [20].
- *Privacy* refers to the level of protection offered to sensitive information in the real data. The synthetic data generation process should not leak information that could be used to re-identify individuals or sensitive details in the original data. Two of the most common types of privacy attacks are the membership inference attack (MIA) and the attribute inference attack (AIA) [27]. MIA occurs when an attacker tries to determine if real patient records have been used to train the synthetic data generative model. In contrast, AIA happens when an attacker has access to some attributes of the real data and tries to guess the value of an unknown patient attribute using synthetic data. Understanding these types of privacy attacks is essential in evaluating the suitability of synthetic data for specific use cases. There are several other privacy measures: differential privacy, different membership attacks (Hyeong et al.), and distance to closest record (DCR), which are used to check the similarity between real and synthetic samples [28].
- The *TabSynDex* is a unified metric for evaluating synthetic tabular data. Unlike traditional methods that rely on multiple metrics, TabSynDex offers a single score to assess the quality of synthetic data. It does not simply average existing metrics. Instead, it calculates sub-scores for different components, which determines the quality of synthetic data, and then combines them into a final score. This allows for a more nuanced understanding of the strengths and weaknesses of the synthetic data. The metric is bound between 0 and 1, making it a valuable benchmark for evaluating the quality of synthetic data. A score closer to 1 indicates better synthetic data quality, considering all the evaluated aspects [14].

In conclusion, synthetic data is evaluated in terms of resemblance/fidelity, utility, and privacy. It is crucial to balance all three of these measures. However, resemblance/fidelity measures are essential when the primary goal is to maintain the authenticity of the real data, that is, preserving its statistical properties and distributions. Utility measures are essential when the dataset is intended for specific analysis, which includes training machine learning tasks, ensuring that the generated data remains valuable and informative for deriving meaningful insights. Privacy evaluation measures are the most important when protecting sensitive information and safeguarding individuals’ identities. Privacy measure ensures compliance with privacy regulations. When the purpose of synthetic data is for data augmentation (increasing the size of real data by adding synthetic samples), measuring privacy might not be necessary.

## Using generative AI for medical applications

Integrating AI into physiology improved the landscape of medical research and patient care, particularly through the transformative applications of generative AI [8]. Key applications, such as data augmentation, improved decision-making, and personalized medicine, will be discussed now to demonstrate the profound potential of AI to enhance and personalize healthcare (Fig. 1). In particular, generative AI models are becoming increasingly important tools in data augmentation and imputation, helping address the limitations of small or incomplete datasets in clinical and physiological research. These techniques enable synthetic data generation to enhance model training, improving decision-making by allowing more robust analyses. Moreover, generative AI facilitates better data collaboration across institutions by creating secure, anonymized datasets for multi-center studies. Such advancements refine predictive models in patient care and push the boundaries of physiological research.

### Data augmentation and imputation for improved decision-making

Artificial clinical trial generation, while primarily applied in fields such as oncology, also holds significant potential for physiology research. AI-driven approaches to clinical trials, including data augmentation and trial design, can be instrumental in exploring physiological phenomena across a broader spectrum of diseases and conditions [13]. By creating artificial trial scenarios, researchers can model complex interactions within the human body, enabling a deeper understanding of physiological processes. For example, studies by Kim and Quintana [32], Haddad [25], and Beck [5] have demonstrated that AI-based systems can efficiently and reliably screen cancer patients for clinical trial eligibility with a high degree of accuracy. This innovation streamlines the patient selection process and ensures that patients are matched with the most appropriate and potentially beneficial trials, enhancing the overall effectiveness of cancer treatment and research. In addition to patient screening, AI is harnessed to improve clinical trial design. Zhang [68] explored how AI can create more efficient, accurate, and patient-centric clinical trials. By analyzing large datasets and identifying patterns that might not be apparent to human researchers, AI can help design more targeted trials, reducing costs and increasing the likelihood of successful outcomes. A recent study by Eckardt [18] delved into generative artificial intelligence, specifically its application in mimicking clinical trials for acute myeloid leukemia patients. This approach allows researchers to generate synthetic patient data closely resembling real-world scenarios by taking into account several resemblance/fidelity, utility, and privacy measures. Such synthetic datasets can be invaluable when real patient data is scarce or difficult to obtain, thus accelerating research and development in critical areas of medicine.

In healthcare and medicine, a *digital twin* can simulate a patient’s physiology, disease progression, or treatment response based on real-time data and historical information [62]. Digital twins, combined with advanced data imputation and augmentation techniques, can improve drug discovery by enabling more accurate simulations of clinical trials. These technologies allow researchers to model patient responses, fill in gaps in incomplete data, and enhance trial designs, ultimately accelerating the drug development process and improving the likelihood of successful outcomes.

A current study by Bordukova et al. [9] underscores the significant role of generative AI in empowering digital twins within drug discovery and clinical trials. In a similar vein, Chakraborty et al. [12] delve into how AI-enabled clinical trials can offer a faster way to conduct research, especially in response to global health crises like pandemics. Drawing lessons from the COVID-19 era, this research suggests that AI can expedite the trial process, enabling rapid responses to emerging health challenges. But how can these virtual trials be reliably utilized in practice? In the work of Subbiah et al. [54], the authors shed light on the growing significance of synthetic or external control arms in clinical trials. These control arms, often based on real-world data (RWD), provide a viable alternative to traditional control groups, particularly in scenarios where conventional trials may be impractical or unethical. In particular, the ARROW trial (NCT03037385), which evaluated Pralsetinib for RET fusion-positive NSCLC, is a notable example of this approach [45]. By using RWD cohorts as an external control arm, the study demonstrated the effectiveness of Pralsetinib, suggesting its potential as a first-line treatment. Moreover, synthetic control arms are increasingly being recognized for their role in drug approvals, especially in the context of rare diseases. The case of selumetinib for pediatric neurofibromatosis exemplifies this trend, where synthetic control arms provided comparative effectiveness analysis that could soon become a primary source of evidence for drug approvals. However, challenges remain, particularly concerning the quality and completeness of data used in synthetic control arms and uncertainties about the appropriateness of external control data. These issues are being addressed through methods like quantitative bias analysis [57].

In summary, the current trajectory of generative AI in medical applications is promising, offering innovative solutions that enhance clinical trial processes, from patient screening to trial design and using digital twins. These advancements not only streamline research and development in medicine but also hold the potential to significantly improve patient outcomes, especially in areas like oncology and hematology, and align ethical approaches to drug discovery and clinical trials.

### Collecting and sharing data more responsibly and freely—a new avenue through AI

Existing barriers to medical data sharing could be overcome through AI-driven data generation technologies, which focus on creating high-quality, privacy-conscious synthetic patient data. These advancements not only protect patient confidentiality but also open new avenues for research. From generating synthetic patient data for causal effect estimation to producing comprehensive genomic and cancer-specific datasets, these technologies enable researchers to explore complex treatment dynamics and genetic patterns without compromising privacy. In 2021, Toi et al. [58] already provided an article about “Next-Generation Clinical Trials and Research with Successful Collaborations,” which highlights not only the importance of AI in modern clinical trials but also the crucial role of collaboration and trust. This perspective emphasizes that integrating technology with strong collaborative networks can significantly enhance the effectiveness and scope of clinical research. A recent studfy of Shi et al. focuses on generating synthetic patient data that respects privacy while allowing for the estimation of causal effects from multiple treatments [53]. Such an approach is particularly valuable in understanding complex treatment dynamics without compromising patient confidentiality. Similarly, Wharrie et al. developed a tool called “HAPNEST,” which enables researchers to conduct extensive genomic studies on synthetic data while maintaining strict privacy protections [63]. Additionally, Scandino’s 2023 research introduces “Synggen,” a method designed for rapidly generating synthetic heterogeneous Next-Generation Sequencing cancer data [50]. By providing realistic and complex datasets, this innovation accelerates cancer research without the ethical concerns typically associated with handling real genomic patient data.

Creating and sharing comprehensive longitudinal datasets is another important aspect in advancing medical research, especially in tracking patient health over time. Mosquera et al. recently introduced a novel method for generating synthetic longitudinal health data, which provides a temporal perspective vital for in-depth analyses of patient health across various time points [40]. This approach is particularly beneficial for studies requiring long-term data without compromising patient privacy. Similarly, Li et al. focused on generating synthetic mixed-type longitudinal electronic health records (discrete and continuous data), which further enhances the realism and applicability of synthetic data for AI-driven health analyses [36]. By producing more detailed and temporally consistent datasets, these innovations open new avenues for longitudinal studies and AI applications in healthcare.

Together, these examples highlight how synthetic data can drive medical research forward while safeguarding privacy and enabling the exploration of intricate biological processes.

### Large language and foundation models in healthcare: a synopsis

LLMs, such as GPT and BERT, are transforming the landscape of medical research by enabling advanced data interpretation and decision-making capabilities. In the field of physiology, LLMs can analyze vast amounts of biomedical literature, clinical data, and patient records to uncover complex patterns that may not be immediately apparent to human researchers. These models offer a high potential for improving personalized medicine by assisting in the prediction of disease progression, tailoring treatments based on individual patient profiles, and enhancing the accuracy of diagnostics [38]. Additionally, LLMs facilitate more informed decision-making in certain domains by integrating diverse data sources, such as genomic, phenotypic, and environmental factors, to provide comprehensive insights that can lead to better patient outcomes [11].

For example, Li [37] and Yu [67] provide comprehensive insights into ChatGPT’s applications and a roadmap for its integration in healthcare. The work of Peng et al. underscores LLMs’ potential in medical research [44]. The notable experiment with GatorTronGPT demonstrated its ability to match human physicians in language and clinical relevance, as reported in a physicians’ Turing test. Tang [56] explores LLMs in synthetic data generation for clinical text mining, showcasing their utility in enriching medical datasets.

Foundation models have recently been introduced as the logical next step to form generalist medical AI (GMAI) applications [39]. In a nutshell, GMAI models will be adept at performing a wide range of tasks with minimal or no task-specific labeled data. Developed through self-supervision on large, diverse datasets, GMAI could flexibly interpret various combinations of medical modalities, including imaging data, electronic health records, laboratory results, genomics, graphs, and medical texts. These models are currently under extensive investigation and already generate expressive outputs, such as free-text explanations, spoken recommendations, and image annotations, showcasing advanced medical reasoning abilities [24, 33]. As these models continue to evolve, their role in medicine and physiology research is expected to expand, offering new avenues for discovery and innovation in healthcare.

## Conclusion

In conclusion, this review highlights the research on tabular generative models and their applications in physiology, emphasizing the challenges of creating high-quality synthetic tabular data. It explores advancements in generative AI, particularly in medical applications, and discusses methods for evaluating the quality of synthetic data. Additionally, the review examines the progress in LLMs within healthcare research, showcasing their potential to improve diagnostics, personalize treatments, and enhance patient outcomes. These developments showcase the impact of AI in medicine and present promising opportunities for future research to advance these technologies further and address current obstacles.

## Data Availability

No datasets were generated or analyzed during the current study.
